# Application of Enzymatic Extracts from a CALB Standard Strain as Biocatalyst within the Context of Conventional Biodiesel Production Optimization

**DOI:** 10.3390/molecules22112025

**Published:** 2017-11-21

**Authors:** Carlos Luna, Diego Luna, Felipa M. Bautista, Rafael Estevez, Juan Calero, Alejandro Posadillo, Antonio A. Romero, Enrique D. Sancho

**Affiliations:** 1 Department of Organic Chemistry, University of Cordoba (UCO), Cordoba 14014, Spain; qo2luduc@uco.es (C.L.); qo1baruf@uco.es (F.M.B.); q72estor@uco.es (R.E.); p72camaj@uco.es (J.C.); qo1rorea@uco.es (A.A.R.); 2 Seneca Green Catalyst S.L., Rabanales XXI/University of Cordoba, Cordoba 14014, Spain; seneca@uco.es; 3 Department of Microbiology, University of Cordoba, Campus de Rabanales, Ed. Severo Ochoa, 14014 Cordoba, Spain; edsancho@uco.es

**Keywords:** biodiesel, ecodiesel, lipases, CALB, enzymatic extracts, transesterification, glycerol

## Abstract

The application of biocatalysts in the transesterification process of triglycerides (TG) allows integrating the glycerol in the form of monoglyceride (MG), sharply increasing the yield and the environmental sustainability of the conventional biodiesel production process. This is known as Ecodiesel. To overcome the inconvenient of the high cost of the currently employed highly purified commercial enzymes, the use of scarcely purified extracts obtained from standard strains of the same species of commercial lipases currently applied in this process is being investigated. Thus, *Candida antarctica* type B (CALB) was chosen to determine the optimal conditions of culture of this yeast. The standard strain was obtained from the Spanish Type Microbial Cultures Collection (CECT) and has been used to carry out several studies to elucidate its optimum growth conditions. Through a process of lyophilization with prior dialysis of the liquid cultures, the enzymatic extracts were obtained. The different obtained cultures have been applied as biocatalysts in the 1,3-selective transesterification reaction of sunflower oil with ethanol to obtain Ecodiesel (FAEE + MG). Selectivity and reaction yields were obtained by gas chromatography. Acceptable yields are obtained during the reaction time as well as in successive reactions, demonstrating the feasibility of using these CALB enzymatic extracts as biocatalysts.

## 1. Introduction

Biodiesel has emerged as an environmentally-friendly and renewable alternative to petroleum-based fuels. Reserves of conventional petroleum-based fuels, which are only located in certain parts of the world, are rapidly diminishing. Because of ever increasing prices of fossil diesel and environmental concerns due to emission of toxic compounds upon its combustion, many countries across the world are encouraging the use of biodiesel as a transport fuel [1]. It is commonly accepted that biofuels are not currently able to fully replace the global demand for fuels, but they can cover an increasing part of them, which greatly reduces the dependence on fossil fuels, prolonging the life of the existing oil reserves, thus softening the transition to a predictable world scenario without fossil fuels [2,3].

Biodiesel is defined as a mixture of monoalkyl esters of long chain fatty acids (FAME) derived from renewable lipid sources, such as vegetable oils or animal fats, that can be used in compression ignition engines, with little or even no modifications [2,3,4,5]. The most usual method to transform oil into biodiesel is a transesterification process that can be carried out using different catalytic systems [6] or under supercritical conditions [7]. The catalysts currently studied for biodiesel production can be grouped in four categories: alkali or acids, homogeneous catalysts, heterogeneous inorganic solid catalysts and enzymes [8]. In the last decade, particular attention has been paid to the use of lipases as biocatalysts for biodiesel production [9]. The pros and cons of using lipases as biocatalysts for biodiesel production compared with alkaline and acid catalysts are summarized in Table 1 [8].

In general, lipases perform their catalytic activity under milder conditions and with a higher variety of triglyceride substrates, including waste oils and fats with high levels of free fatty acids (FFA). Furthermore, biodiesel separation and purification is much easier, resulting in a more environmentally friendly process.

Lipases are found in animals, plants and microorganisms and play a key role in the metabolism of oils and fats. In addition. lipases take part in the deposition, transfer and metabolism of lipids [10]. Lipases are hydrolases (EC 3.1.1.3) that act on carboxyl ester bonds in triglycerides to yield fatty acids and glycerol. Lipases catalyze this reaction at the lipid–water interface. Lipases have both hydrolytic as well as synthetic activity and, thus, can participate in various industrially important reactions such as esterification and transesterification (alcoholysis and acidolysis). Lipases from fungi and bacteria are easily produced in bulk amounts because of their extracellular nature [11]. Lipases currently are widely used in the processing of fats and oils, detergents and degreasing formulations, food processing, the synthesis of fine chemicals and pharmaceuticals, paper manufacture, and production of cosmetics [12]. These enzymes have also excellent catalytic activity and stability in non-aqueous media and their specificity, regioselectivity and enantioselectivity can be successfully used for many applications in organic synthesis, including kinetic resolution and asymmetric synthesis [8]. In terms of regioselectivity, lipases have been divided in three types: sn-1,3-specific (hydrolyze ester bonds in positions R1 or R3), sn-2-specific (hydrolyze ester bond in position R2) and nonspecific (do not distinguish between the positions of the ester bonds to be cleaved). Most known lipases are 1,3-regiospecific with activity on terminal positions. Another important aspect to consider is the acyl migration phenomenon inside the triacylglycerol molecule, reported in several studies [13].

Commercially produced lipases are mostly of microbial origin. Submerged culture and solid-state fermentation are the most widely used methods for commercial lipase production. Lipase-producing microorganisms such as bacteria, fungi and yeasts are isolated and screened to determine their lipolytic activities [11,14]. Based on the lipolytic activity, microorganisms are then chosen for commercial lipase production. Lipase production depends on many factors such as carbon and nitrogen source, pH, temperature, dissolved oxygen, agitation and metal ions. Lipase production can also be induced by providing lipids as a carbon source [15]. The purification strategy includes concentration of the culture medium by ultrafiltration or ammonium sulphate precipitation followed by further purification using sophisticated techniques such as affinity chromatography, ion exchange chromatography and gel filtration [11]. Several novel techniques like membrane processes, immunopurification, hydrophobic interaction chromatography or column chromatography are also currently applied for purification of lipases [16]. In any case, the production and purification schemes of lipases, for large scale application, should be high yielding, rapid and inexpensive [8,17].

There are two main categories of enzymatic biocatalysts: extracellular lipases and intracellular lipases. In case of extracellular lipases, the enzymes are previously recovered from the live-producing microorganism broth and then purified, whereas intracellular lipase remains either inside the cell or in the cell producing walls. The major producer microorganisms for extracellular lipases are *Mucor miehei*, *Rhizopus oryzae*, *Candida antarctica* and *Pseudomonas cepacia* [8]. Studies reporting the use of free lipases for biodiesel production have principally focused on screening of lipases [18] and on investigating the factors that influence the reaction rate [19]. Soluble lipases have the advantage of an easy preparation procedure and low cost, but in many cases, they can be used only once, as they are inactivated after the first use. Improved immobilization technologies provide lipases with enhanced levels of reusability and operational stability, resulting in higher efficiency under working conditions [20]. To achieve the most efficiency and of course economic viability, suitable raw materials and lipase preparation methods must be chosen. The latter can be modified to improve stability and catalytic efficiency. These steps are followed by selection of organic solvent, optimization of molar substrate ratio, temperature, water activity, pH of the enzyme’s microenvironment and the highest permissible glycerol concentration in the reaction products (the so called sub-parameters).

To reduce the production cost of enzymatic transesterification, the catalytic stability and activity of lipases can be improved by protein engineering and metabolic engineering techniques [21]. In addition, further reduction of the running cost of enzyme-catalyzed biodiesel production can be achieved by process intensification strategies, such by improving the immobilization or by process design and optimization. Immobilization of lipases has been studied for many years, and various supports have been used [9]. However, only very few types of immobilization process have been commercialized. Enzymatic catalysis in biodiesel production is a relatively new research field, that it is attracting increasing interest from the scientific community and the biodiesel industry [20]. In the recent past, novel techniques have been developed to make enzyme catalysis sustainable and economically viable. These techniques mainly deal with reducing the price of enzymes as well as with improving the efficiency of transesterification conversion.

To avoid the problems associated with the generation of glycerol in the conventional process, a series of alternative methods are currently considered to get the highest atom efficiency. This strategy consists in the production, in only one reaction, of new biofuels that also integrate the glycerol as a derivative product, miscible with the fatty acid methyl or ethyl esters (FAME or FAEE) obtained in the same transesterification process. Basically, this is possible by using some alternative esters, instead of the short chain alcohols usually employed in the conventional process. Thus, if some glycerol derivative compound is obtained together to FAME (or FAEE), in an interesterification process, a new biofuel is obtained in only one reaction avoiding the presence of free glycerol [3,4]. These methodologies avoid the separation of glycerol before its transformation, simplifying the process [22,23]. These biofuels not only prevent the generation of waste, but also increase the yields of the process, always higher than nominal 12 wt %, by incorporating some derivatives of glycerol into the reaction products. Besides, every one of the reactants employed in excess, remains in the reaction products blend as a part of the obtained biofuel, so that the highest atom efficiency, practically 100 wt %, is obtained. Novel methodologies to prepare esters from lipids using different acyl acceptors which directly afford alternative co-products are currently under development [24,25,26].

The interesterification processes can be performed with the same catalysts applied in transesterification processes (homogeneous or heterogeneous, acid or basic catalysts, lipases, supercritical conditions, etc.), although recently the majority of the processes for biofuels production, are being developed using different lipases [22,24] where, instead of using methanol, the lipase-catalyzed synthesis of fatty acid alkyl esters can also be performed using alternative alcohol donors such as methyl or ethyl(alkyl)acetate and dimethyl or diethyl carbonate. These mixtures including glycerol derivative molecules have relevant physical properties to be employed as novel biofuels. Even the used reactants remain together with obtained reaction products and are capable to directly be employed as biofuels [24,25,26,27]. In this way, mixtures of fatty acids methyl esters (FAMEs) and glycerol triacetate (triacetin) are the main products of the interesterification reaction of triglycerides with methyl acetate, and all these products can be used as components of a new type of patented novel biofuel, named Gliperol^®^ that exhibits fuel characteristics comparable to conventional biodiesel fuel [27]. Dimethyl carbonate (DMC) can be used also as a transesterification reagent for making esters from lipids which directly yield alternative soluble co-products in the biodiesel solutions. The reaction is rather attractive, as DMC is reputed to be a prototypical green reagent due to its health and environmental inertness [28]. The reaction between triglycerides and DMC produces a mixture of FAMEs and cyclic fatty acid glycerol carbonate esters FAGCs, which constitutes a novel biodiesel-like material, named DMC-BioD^®^ in the corresponding patent [29].

**Scheme 1:**
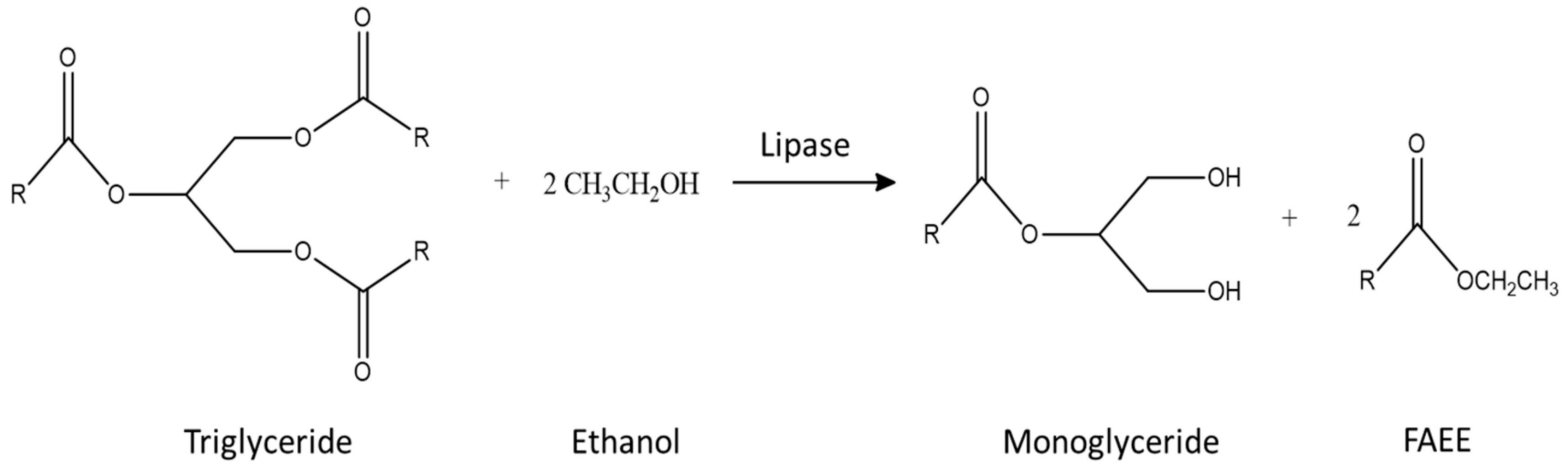
Ecodiesel^®^-100 is a biofuel obtained by enzymatic technology patented by University of Cordoba (UCO) incorporating glycerol, as it is formed of two moles of ethyl esters of fatty acids (FAEE) and one mole of monoglyceride (MG).

There is another innovative proposal of this approach integrating glycerol derivatives in the biofuel, developed by our group, consisting in a biodiesel-like biofuel produced by obtaining monoacylglycerol in the same transesterification process of oils and fats. In this respect, a protocol for the preparation of a new kind of biodiesel that integrates glycerol into their composition via 1,3-regiospecific enzymatic transesterification of sunflower oil was developed, using pig pancreatic lipase (PPL) soluble [30,31] as well as in an immobilized way [32,33]. The procedure takes advantage of the 1,3-selective nature of lipases, which allows one to “freeze” the process in the second step of the alcoholysis by obtaining a mixture of two moles of FAEE and one of MG, as can be seen in Scheme 1. This strategy is based on the obtention of an incomplete alcoholysis by application of 1,3-selective lipases, so that the glycerol remains in the form of monoglyceride which avoids the production of glycerol as by-product, reducing the environmental impact of the process. Thus, the already patented Ecodiesel^®^-100 [34], is a mixture of two parts of FAEE and one part of MG, that integrates the glycerol as a soluble derivative product (MG) in the diesel fuel. But unlike these methods, no special reagent, more expensive than ethanol, such as dimethyl carbonate or methyl acetate, is used. Table 2 shows a summary the pros and cons of the different existing methodologies for obtaining biofuels integrating glycerol, as a derivative able to work as combustible, together to FAME or FAEE, thus avoiding the presence of free glycerin [4].

Ecodiesel^®^ not only exhibits similar physicochemical properties to those of conventional biodiesel, but also monoacylglycerides (MG) were proven to enhance the lubricity of the biodiesel as recent studies have demonstrated [35]. Besides, ethanol not spent in the enzymatic process can remain in the reaction mixture in such a way that the obtained product blend can be directly used as a fuel. In this respect, some studies [36] have proven that blends of diesel fuel and ethanol with biodiesel only produce a slight decrease in maximum power outputs, respect to regular diesel. Besides, no significant difference in the emissions of CO_2_, CO, and NO_x_ between regular diesel and biodiesel, ethanol and diesel blends were observed, and the use of these blends resulted in a significant reduction of particulate matter. Consequently, such blends can be used in a diesel engine without any modification, considering the limited changes obtained respect to the use of pure diesel. Thus, the term Ecodiesel^®^ is currently ascribed to any blend of fatty acid alkyl ester with ethanol, alone or with any proportion of diesel fuel [37,38,39].

Although Ecodiesel^®^ was initially developed by using porcine pancreatic lipases (PPL), later several studies have been carried out to identify lipases that are more viable from an economical point of view. In this respect, remarkable results have also been obtained with the microbial lipase Lipopan 50 BG (Novozymes AS, Kalundborg, Denmark) [40], a low cost purified lipase from the microorganism *Thermomyces lanuginosus*, usually used as a bread emulsifier (bread improver) [41]. Enzymatic ethanolysis reaction of sunflower oil with ethanol, in free solvent media, has also been studied by using Biolipase-R, a multipurpose alimentary additive from Biocon^®^-Spain (Barcelona, Spain) that is a low-cost lipase from a strain of *Rhizopus oryzae* [42]. A commercial recombinant lipase from *Candida antarctica*, expressed in *Aspergillus niger*, Novozym 435 [43], as well as Lipozyme RM IM, a *Rhizomucor miehei* lipase immobilized on a macroporous anion exchange resin have also been evaluated in this selective transesterification process [44]. A *Rhizopus oryzae* lipase, from Biolipase-R, immobilized on Sepiolite, an inorganic support, was similarly studied to examine the economic viability of the procedure [45].

In a recent work some microbial lipases have been selected from among several wild microorganism strains found in several lipophilic environments, like in an olive oil press, or in animal fats [46]. It was observed that the best two performing strains belong to the genus *Terribacillus*, which has not been reported as a biofuel producer so far, but it was also noted that the enzymatic extracts from these microbial strains were obtained after a minimum purification treatment, through a lyophilization process with a previous dialysis treatment. This fact suggests a novel alternative for synthetic processes because it has thus been shown that an extract of very poorly purified lipases can be used to produce a cheap enzymatic extract, able to produce cheaper biofuels by reducing the cost of enzyme production.

In the present research study, it is assumed as a new paradigm, that one can use very little purified enzymatic extracts of lipases, as a procedure to reduce the operation costs of the transesterification process. Thus, in the current study we proposed to obtain enzymatic extracts from a standard strain of the same species previously evaluated as commercial enzymes [43], so *Candida antarctica*, (CA), one of the most common and well known yeast lipase producers was chosen [8]. Besides, this *Candida antarctica* lipase B (CALB) is usually applied to obtain biodiesel or Ecodiesel^®^. In this respect, a standard strain purchased from the Spanish Type Culture Collection (CECT) where these extracts were obtained from broth growths, by lyophilization with a prior dialysis filtration was used. This is a simpler (and cheaper) purification method than the highly purified commercial systems. Thus, we try to show the advantages of using cheap lipase extracts, instead of conventional purified lipases, opening a new way to deal with the production of alternative biofuels using an enzymatic approach, which is both technically and economically viable.

## 2. Results

Taking into account that the overall objective is the identification of biocatalysts capable of performing enzymatic ethanolysis in an economically viable way, the possibility of employing enzymatic extracts that have not been exhaustively purified has been explored. In this way, the economic costs would be much lower than those of the available commercial enzymatic preparations, which are much more purified, but also quite more expensive. To this goal, a standard *Candida antarctica* yeast strain of, purchased from CECT, that produces lipase B (CALB) has been used to perform several studies in order to elucidate the optimum growth conditions for this strain [47,48,49]. The different cultures obtained have been tested as biocatalysts in the transesterification reaction of sunflower oil with ethanol under standard reaction conditions to obtain Ecodiesel^®^ (FAEE + MG).

### 2.1. Measurement of the Standard Candida antarctica Strain Growth in Different Broth Media

The growth evolution of this CA standard strain obtained from the CECT, along the growth time, using the different studied media [47,48,49], has been determined from measurements of absorbance at 600 nm.

**Figure 1:**
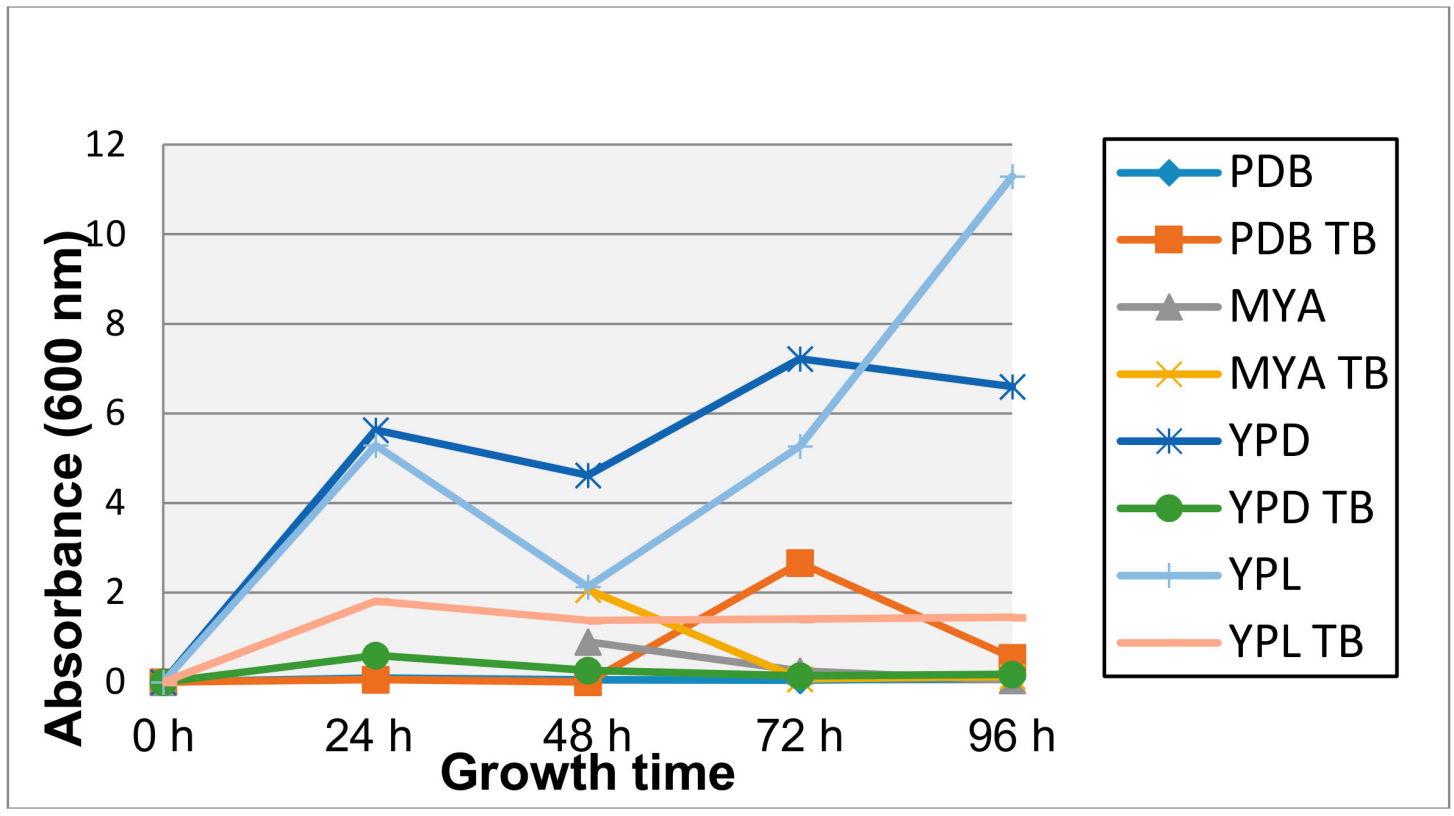
Growth evolution of the *Candida antarctica* standard strain from the CECT, in different growth media, with and without tributyrin (TB), obtained every 24 h through absorbance measurements at 600 nm.

The obtained results are shown in Figure 1, where it can be concluded that the most appropriate growth media are YPD and YPL (better without tributyrin). Besides, the optimal growth time is at 72 h, when there is a maximum peak, as later the growth decreases.

### 2.2. Performance of the Enzymatic Extracts Applied as Biocatalysts in the Transesterification Reaction

**Figure 2:**
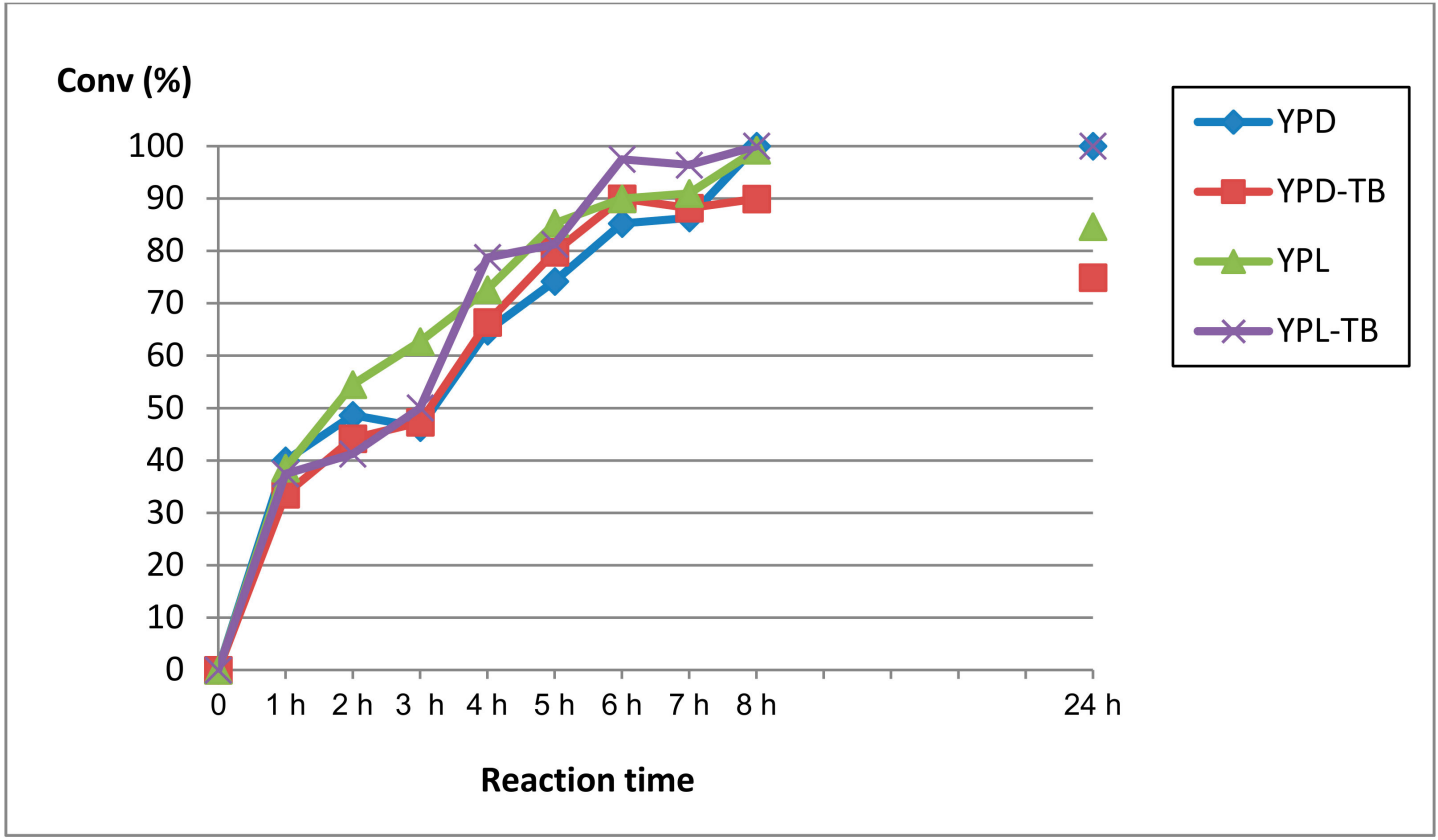
Yield evolution of the selective transesterification along the reaction time, operating under standard reaction conditions: 6 mL of sunflower oil, 1.75 mL of ethanol, 30 °C, 25 μL of 10 N solution of NaOH, higher stirring than 300 rpm, along 24 h as reaction time. As a biocatalyst, 0.5 g of enzymatic extract of the standard CA strain from CECT, obtained from several extracts of YPD and YPL, with and without tributyrin, after 72 h of growth time, are employed.

For the study of the selective transesterification reactions of sunflower oil with ethanol, to obtain Ecodiesel^®^ biofuel (FAEE + MG), extracts of YPD and YPL, with and without tributyrin, the most appropriate culture media as well as the optimal growth time (72 h) were selected [46]. Experimental reaction conditions were the same previously used with other enzymatic systems (6 mL of oil, 1.75 mL of ethanol, 30 °C, 25 μL of a 10 N NaOH solution, agitation around 300 rpm and 24 h of reaction time) [40,42,43,44,45]. The conversion values evolution of the selective transesterification reaction along the reaction time, operating under standard reaction conditions are collected in Figure 2. As biocatalyst, 0.5 g of enzymatic extract from the standard CA strain of the CECT, obtained from several growth broths are employed.

To confirm the reproducibility of the global procedure developed to obtain the extracts of the different investigated cultures, a second reaction series was obtained by applying as accurately as possible the conditions of culture, extraction and purification of the extracts applied as biocatalysts in the selective transesterification process (Table 3). Furthermore, the results of the reactions performance without biocatalyst or without inoculum, and consequent growth of the standard microbial strain, as blank biocatalysts are also shown. Thus, as blank reaction the results obtained when operating without any extract, or with the medium of an extract, without inoculating any strain are considered. To assess the influence of the enzymatic process, the comparative values of pure sunflower oil are indicated. These results in Table 3 confirm the catalytic action of the lipase enzymatic extracts obtained of the standard CA strain from the CECT, after being cultivated in the selected media of YPD and YPL, with and without tributyrin.

The ultimate objective intended when subjecting an oil or fat to a transesterification or alcoholysis process is to modify its rheological properties, mainly to reduce its viscosity, in order to it can be used in diesel engines, without any further modification. For this reason, to obtain information of the efficiency of the enzymatic process, it is necessary to compare the viscosity of the sunflower oil with those of the obtained products, after the development of the studied enzymatic process. Thus, considering that the selectivity values refer to the amount of FAEE and MG, compounds of lower viscosity, the high viscosity of sunflower oil is due not only to the high amount of TG, but also to the presence of DG, according to the parameter measured in Conversion. This last parameter refers to all the products obtained in the transesterification reaction, i.e. FAEE, MG and DG. The presence of ethanol, as a reagent that has not been completely spent in the alcoholysis enzymatic process, also brings an important contribution to the reduction in the viscosity of the reaction products. It is also interesting to point out that the presence of tributyrin (TB) in the culture medium does not bring greater efficiency to the lipases obtained, which is verified in a slight decrease in the values of Conversion and Selectivity values obtained.

### 2.3. Study of the Activity of the Enzymatic System in Succesive Reactions

**Figure 3:**
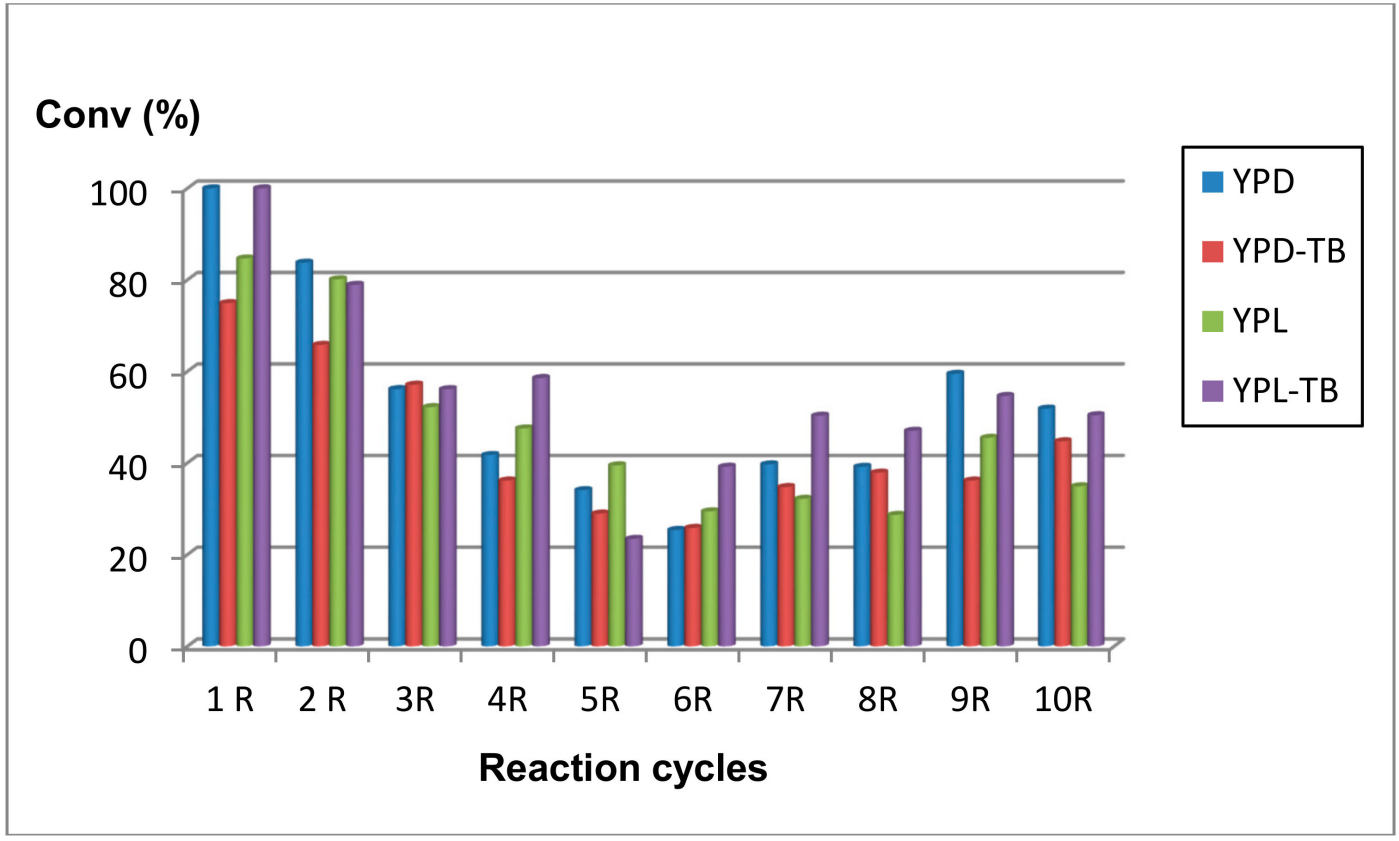
Yield of the selective transesterification reaction in successive reuses of the same biocatalytic system, under standard reaction conditions: 6 mL of sunflower oil, 1.75 mL of ethanol, 30 °C, 25 μL of 10 N NaOH solution, stirring higher than 300 rpm, during 6 h as reaction time. As a biocatalyst is applied 0.5 g of enzymatic extract from a standard CA strain purchased in the CECT, obtained from several growths broths.

Since the possibility of reuse any catalytic system is an essential parameter, a series of experiments have been performed with consecutive reactions under the same experimental conditions, using the same biocatalytic system, to verify its stability and vigorousness. Thus, applying identical experimental conditions to those shown in Figure 2 and Table 3, 0.5 g of enzymatic extract of the standard CA strain of the CECT, obtained from a CA broth with the different growth media were employed. In this series of consecutive reactions, the reaction time is reduced to 6 h, since it is the time where optimum levels of performance are reached. The results obtained are shown in Figure 3.

These results show that the lipases extracted, together with other enzymes, since they are extracts subjected to a very elemental purification, maintain their activity quite consistently, since it is expected that the decrease in activity is mainly determined by the loss of the enzymatic material in the manipulation of the extraction process of the reaction products. This loss of enzymatic material is appreciable because it is carried out by decanting the reaction product. Thus, with this extracting from the reaction bottle, a part of the enzymatic material is also lost. However, the method used simply aims to confirm that the enzymes maintain enough activity after reuse. This would allow the implementation of the procedure in its industrial application, so that the loss of enzymatic material during the reuse was minimal.

## 3. Discussion

According to the results here obtained, the feasibility of these CA enzymatic extracts as biocatalyst is demonstrated. In this respect, once the extracts were obtained in the optimum media (YPD and YPL), both with and without tributyrin (TB), and with the determined optimal growth time (72 h), in all tested reactions, enough effective yields are obtained along the reaction time, to get after 6 h the optimal conversion values. This demonstrate their biotechnological potential and technical presumed viability at an industrial level. Then, its use could make considerably more sustainable the Ecodiesel^®^ production from an economical point of view. Thus, using a very simple methodology of extraction and purification of the enzymes from a growth broth, consisting of a lyophilization with previous dialysis, it is possible to access to an enzymatic material, capable of obtaining not only the minimum requirements to carry out the process of enzymatic alcoholysis, but also results better than those obtained with more purified and much more expensive commercial lipases. Consequently, it is clearly advantageous to produce Ecodiesel^®^ using lipase extracts obtained from growth broths, in a simple and economical way, instead of using the commercially available purified lipases, such as PPL [30,31,32,33,34], Lipopan 50 BG [40,44], Biolipasa R [42,45], Novozym 435 [43], Lipozyme RM IM [44], all of which were previously studied to determine the economic viability of the procedure.

In addition, according to the results of the yield studies throughout their reaction time (Figure 2) and in successive reactions (Figure 3) of the extract from the standard strain of *Candida antarctica*, as a free-form biocatalyst, in the ethanolysis reaction of sunflower oil, operating under the same standard experimental conditions, the ability of these extracts to be reused can be clearly concluded, which represents a very positive aspect for the possible application of these enzymatic extracts, free or immobilized, in any conventional support.

These enzymatic materials are easily obtained from liquid culture growths, with a very simple purification technique, and are presented as optimal biocatalysts with very low cost of production, since they have not been subjected to any additional high cost purification treatment, like chromatography or electrophoresis, generally applied to obtain the usual commercial purified enzymes. Therefore, 0.5 g of these materials can provide an enzymatic activity equivalent to that of 0.01–0.04 g of a commercial lipase, much more difficult and also more expensive to obtain, so that in this poorly purified enzymatic material, there is enough amount of lipase capable of carrying out selective ethanolysis reactions of triglycerides to obtain the mixtures of FAEE and MG constituting the Ecodiesel^®^ biofuel.

As the main result, these low-cost lipase extracts are especially efficient in the production of selective ethanolysis processes, where glycerol is maintained as MG in the biofuels mixture, together with the different FAEEs obtained and the excess ethanol without reacting. These reaction product mixtures, which may also contain small amounts of unreacted diglycerides and triglycerides, constituting in this way, a new type of biodiesel called Ecodiesel^®^, that can be used in different blends with diesel fuel, without further separation or purification process. This biofuel can also be obtained in relatively short reaction times (6 h) and under mild reaction conditions. In addition, an optimum atomic yield (100%) is achieved because glycerol is not generated as a by-product, so it is not necessary to perform a purification step of the residual glycerol, so that it could be used directly after its production. Therefore, these results show the viability of a new paradigm related to the application of enzymes in fine chemistry, which consists of using scarcely purified extracts, which should drastically reduce the price of enzymatic extracts capable of acting as a biocatalyst.

## 4. Materials and Methods 

Commercial sunflower oil was locally obtained. The chromatographically pure ethyl esters of palmitic acid, stearic acid, oleic acid, linoleic acid, and linolenic acid were commercially obtained from AccuStandard (New Haven, CT, USA), and hexadecane (cetane) was obtained from Sigma-Aldrich (St. Louis, MO, USA). Other chemicals like absolute ethanol and sodiuesm hydroxide were pure analytical compounds (99.5%) obtained commercially from Panreac (Castellar Del Valles, Spain).

### 4.1. Obtention and Optimization of the Biocatalytic System

In this section are described several processes carried out to elucidate the optimum growth conditions of the CA standard strain and the whole process to obtain the biocatalytic system [47,48,49].

#### 4.1.1. Characteristics of the CA Standard Strain

After acquiring the strain from the CECT, this institution provides information about the strain [50]. It is specifically numbered as CECT strain 13,054, it was isolated for first time in the Antarctica and its most popular use is like in our case for lipase production. The optimal culture conditions are 20 °C, for 72 h, under aerobic conditions.

#### 4.1.2. Growth Media

The culture media employed were those indicated as optimal by the CECT in its protocol (PDA and MYA), in addition it was decided to test the most common medium for yeasts (YPD) and a variation of it (YPL), with lactose instead of glucose. The growth media were liquids in form of broth or solids (with agar in their composition). After the preparation, they were conveniently sterilized in autoclave at a temperature of 121 °C for 20 min. After its autoclaving, agar media were stored in the oven, to prevent their solidification before being poured into the corresponding plate. Broths media are prepared and stored in Pyrex containers for storage or in flasks (500 mL) for the growth of the CA standard strain (100 mL medium). Agar to generate solid media were hot-poured into petri dishes, where at ambient temperature they solidified (about 25 mL of medium per plate).

To verify the lipolytic activity of this strain and to promote lipase secretion, media supplemented with tributyrin (0.5%) were also used. Tributyrin, being a triacylglycerol, is insoluble in water, so for the elaboration of the medium was necessary to treat it in an ultrasonic bath for at least 10 min at maximum power, in order to obtain an emulsion that homogenizes the medium. A sonicator (SONIFER 250, Branson, Newtown, CT, USA) was used for this purpose [46].

#### 4.1.3. Inoculation and Growth of the C.A. Standard Strain in the Culture Medium

**Figure 4:**
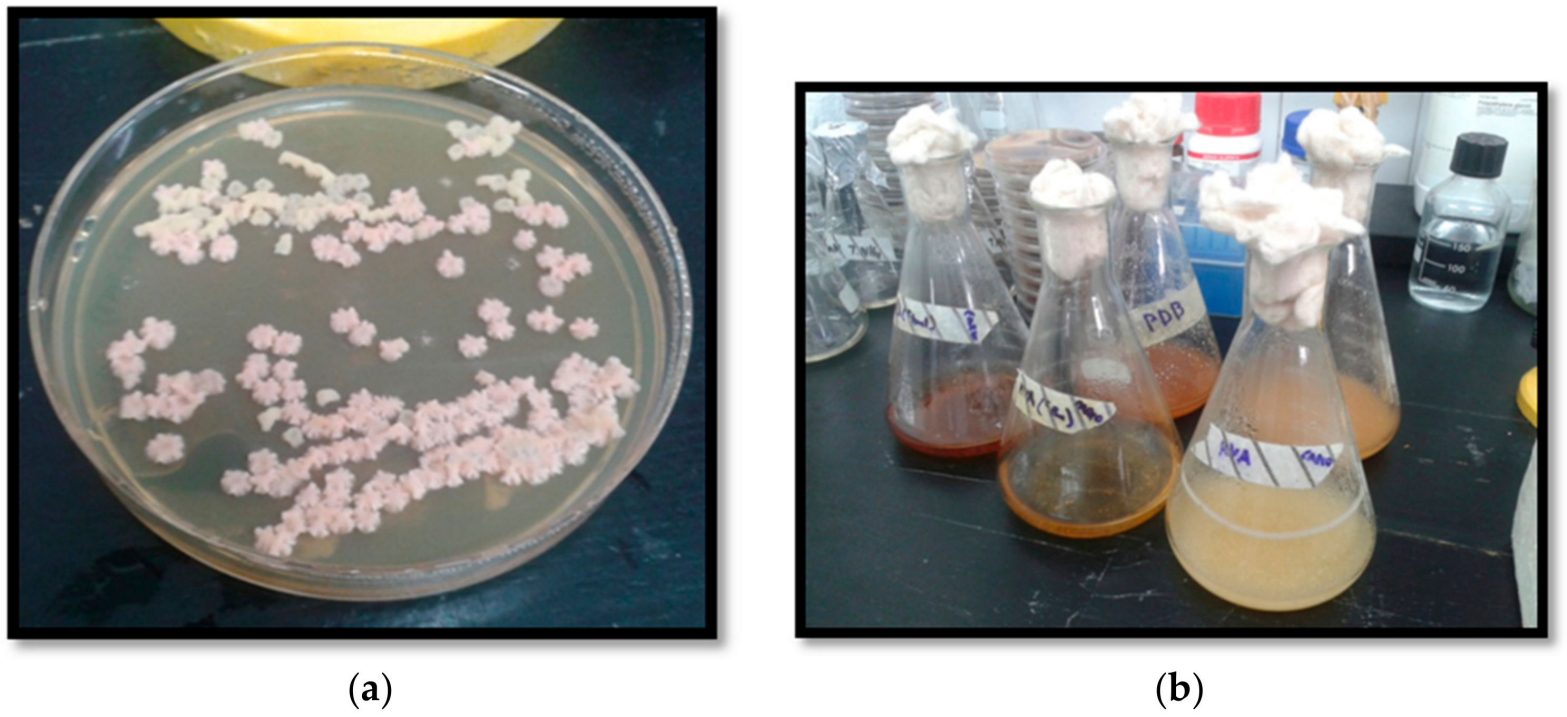
(**a**) Solid culture of the CA standard strain in MYA; (**b**) CA standard strain liquid cultures in different media, PDB, MYA, YPD and YPL.

- Solid medium: from the original tube in petri plates with PDB (Figure 4a), from there, they were re-inoculated in the other plates with the different growth media, MYA, YPD or YPL.
- Liquid medium: A colony was poked and submerged in 10 mL of a liquid medium in a 50 mL tube, it was left its optimum growth time (72 h) under stirring (200 rpm) at 30 °C and then it was passed to a flask in order to be growth under the same conditions, to perform growth studies, to determine its transesterification capacity or to obtain its enzymatic extract, through a dialysis (12 kDa) and a lyophilization during 48 h. It is also possible to accelerate this growth process by pre-inoculating a liquid “preinoculum”, thus leaving growth a chopped colony in a 50 mL tube for 24 h. Then, 500 μL of it were injected into 100 mL of medium in a 500 mL flask (Figure 4b) [46].

#### 4.1.4. Conservation of the Frozen Strain

After the inoculation of the CA standard strain in one of its optimal media, in 10 mL of the medium (PDB, MYA, YPD or YPL) in a 50 mL tube and, after the adequate time to grow (72 h), it was subjected to centrifugation under the following conditions: 4500 rpm, 4 °C, for 5 min. The pellet was resuspended in 600 μL of medium + 400 μL of glycerol (50% *v*/*v*) and placed in a freezing vial. It was subjected to vortexing for a few seconds and stored at −20 °C or −80 °C.

#### 4.1.5. Growth Measure of the CA Standard Strain

Samples were taken (in a different way if they were media without tributyrin or with it) and the absorbance was measured at 600 nm in a DU 640 spectrophotometer (Beckman, Oakley Ct, Windsor, UK). The level of bacterial growth was determined spectrophotometrically since the higher the microbial growth in the liquid culture medium is, more absorbance is measured.

- Cultures without tributyrin: a blank is taken (1 mL of culture medium) and the sample is taken: 900 μL of culture medium + 100 μL of sample (obtained from the culture flask).
- Cultures with tributyrin: 1 mL of sample (from flask with culture) is subjected to centrifugation (1300 rpm, 3 min). The supernatant is then taken as a blank. The sample will be prepared by resuspending the pellet in 1 mL of the corresponding medium (without TB) by vortexing.

#### 4.1.6. Obtaining Enzymatic Extracts from Broths of Standard CA Strains: Lyophilization with Previous Dialysis

**Figure 5:**
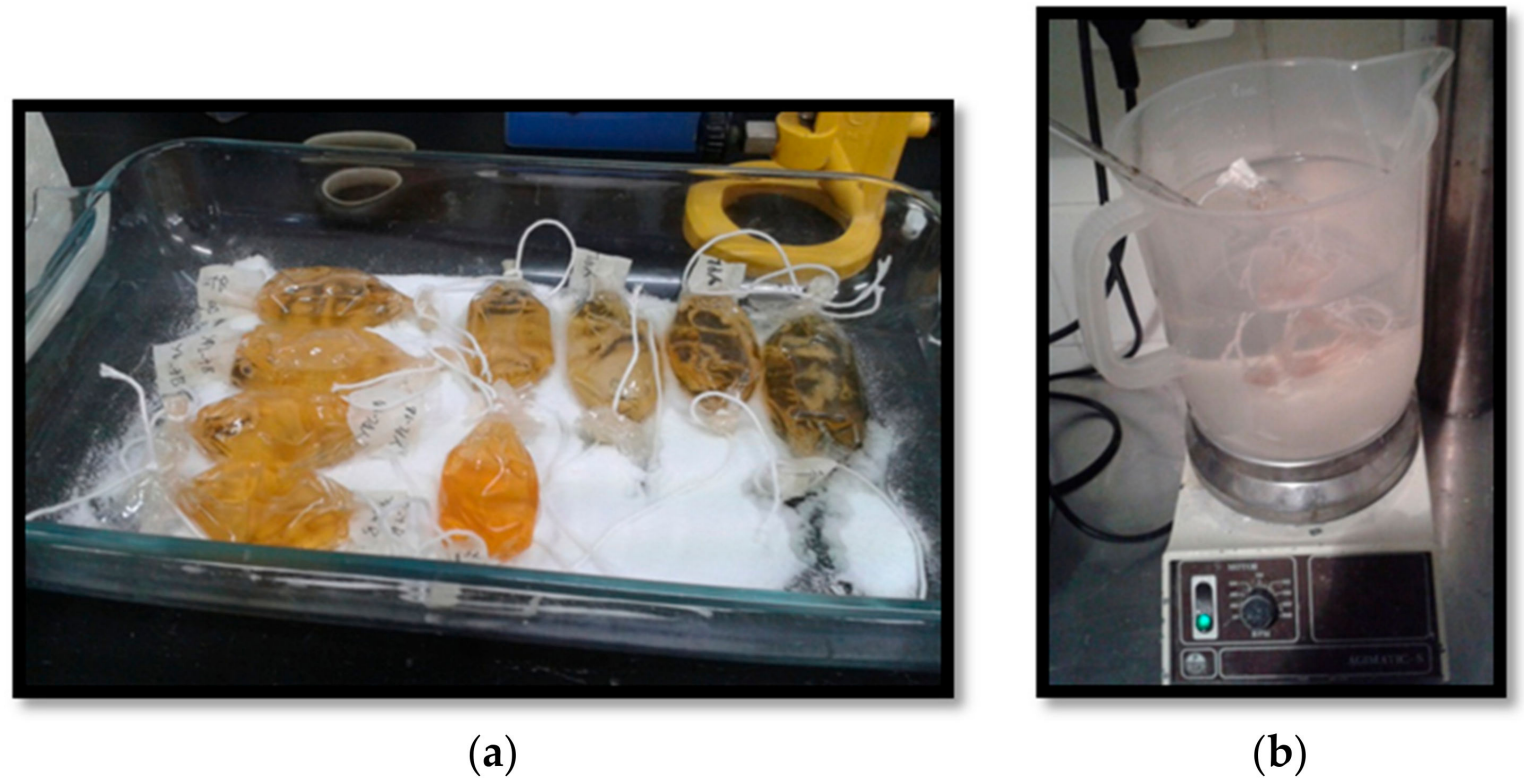
Dialysis process prior to lyophilization to obtain the enzymatic extracts. (**a**) Preparation of the samples for dialysis, with the membranes in characteristic cylindrical form; (**b**) Wash with phosphate buffer dissolution of the dialysis samples.

Through the previous tests of optimization, it was concluded that the most suitable media are YPD and YPL (with and without TB) at 72 h as optimal growth time, under 30 °C and 200 rpm. The dialysis membranes (D-9527, Sigma, St. Louis, MO, USA), with 43 mm of diameter, were placed in hot distilled water for a few hours. Each flask with 100 mL of broth culture, was divided between four Falcon tubes with 25 mL of culture, which were later centrifuged at 4000 rpm, under 4 °C for 10 min. The Falcon tubes were placed in a box with ice, to keep them cold and so to avoid the denaturization of the proteins dissolved in the extracellular fluid (supernatant). We elaborate the samples to be dialyzed with the membranes in the characteristic cylindrical form, with tied little ropes at the ends of the membranes, and then the extracellular liquid of the Falcon tube was poured inside these membranes (Figure 5a). The cylindrical dialysis preparations were placed in a tray covered with PEG, and then covered with polyethylene glycol (PEG 8000), as it is shown in Figure 5a. This tray was also covered with aluminium foil and kept in a cold room (4 °C) for 12 h. The liquid is removed though the membranes by osmotic difference, resulting in the concentration of the extract.

Once the 0.05 M potassium phosphate buffer (pH 7.8) was prepared, the dialyzed cylindrical preparations closed with little ropes, were placed in a big beaker (2 L) with pipettes to wash them with the prepared buffer solution, at 4 °C and under 400 rpm, to be rehydrated (Figure 5b). Two successive washes of one hour each one was performed, changing the buffer each time. The obtained liquid from the dialyzed cylindrical sample were transferred to their corresponding Falcon tube, normally 4–5 mL were obtained, and they were preserved by freezing (−20 or −80 °C). The process of the extract preparation of the final extracts was finished after a lyophilization treatment for 48 h.

### 4.2. Experimental Design: Ethanolysis Reactions

These reactions were performed according to the previously described experimental procedure in order to determine the optimal conditions for obtaining the selective ethanolysis reaction employing commercial CALB and other lipases [31,32,33,40,42,43,44,45,46]. An alkaline environment, amount of lipase, the oil/ethanol molar ratio (*v*/*v*), and temperature are important parameters to obtain the best behaviour of lipase biocatalysts. Thus, enzymatic assays are carried out with 4.7 g (6 mL, 0.005 mol) of commercial sunflower oil at a controlled temperature (30 °C) in a 25 mL round bottom flask. Reaction mixtures were stirred with a conventional magnetic stirrer at a stirring speed higher than 300 rpm to avoid mass transfer limitations, along a reaction time of 6 or 24 h. The influence of alkaline environment values was achieved by adding 25 μL of 10 N NaOH aqueous solution. As a biocatalyst 0.5 g of enzymatic extract from a standard CA strain from the CECT is applied, obtained from several broth cultures. Following the same methodology of preceding studies [46] the enzymatic content was checked, applying the Bradford method, then logically, how this protein content was correlated with the transesterification effectivity of these enzymatic extracts was also probed.

Studies in successive reactions of the ethanolysis reaction were conducted with the selected enzymatic extract, operating under the standard experimental conditions (6 mL of sunflower oil, 1.75 mL of absolute ethanol and 12.5 μL of 10 N NaOH, 300 rpm stirring, at 30 °C for 24 h). The procedure was simple, as the reaction products are extracted with a pipette, after having been centrifuged for 30 min at 1500 rpm. Then, the previously used extract was left in the bottom of the tube where the reagents for generating a new reaction cycle was added.

### 4.3. Characterization of Reaction Products

Reaction products were monitored by capillary column gas chromatography, using a Varian 430-GC gas chromatograph (Varian Inc., Palo Alto, CA, USA), connected to a HT5 capillary column (25 m × 0.32 mm ID × 0.1 μm, SGE, Supelco, Madrid, Spain with a flame ionization detector (FID) at 450 °C and splitless injection at 350 °C. Helium is used as carrier gas with a flow of 1.5 mL/min. A heating ramp from 90 °C to 200 °C at a rate of 7 °C/min has been applied, followed by another ramp from 200 °C to 360 °C, at a rate of 15 °C/min, keeping the temperature of the oven at 360 °C for an additional 10 min. Cetane (*n*-hexadecane) is used as an internal standard, to quantify the content of ethyl esters and the different glycerides (mono-, di-, and triglycerides) with the help of several commercial standard fatty acid esters. This method allows us to perform a complete analysis of the sample in a single injection and during a time not longer than 60 min, which simplifies the process and increases the speed of analysis [42,43,44,45,46]. Considering that sunflower oil is constituted by a mixture of fatty acids (mainly linoleic, oleic, palmitic, and stearic acids) in variable proportions, the results obtained are expressed as the relative amounts of the corresponding ethyl esters (FAEE, fatty acid ethyl esters), monoglycerides (MG), and diglycerides (DG) that are integrated in the chromatogram. The amount of triglycerides (TG) which has not reacted is calculated from the difference to the internal standard (cetane). Thus, the Conversion includes the total amount of triglyceride transformed (FAEE + MG + DG) in the ethanolysis process, and Selectivity refers to the relative amount of FAEE + MG obtained. The latter are the ones having retention times close to the cetane standard, which is the reference hydrocarbon for conventional diesel fuel.

#### Viscosity Measurements

The transesterification reactions of oils and fats are necessary for its transformation into biodiesel in order to obtain an important reduction in the viscosity of these materials, as they share with the conventional fossil diesel similar values in all chemical-physical significant parameters, except the viscosity. In this respect, most vegetable oils exhibit viscosity values in the range 30–45 cSt, while the fossil diesel is in the range 2.5–6 cSt. Thus, due to the importance of viscosity for the correct running of diesel engines, this parameter becomes a critical factor to change the chemical-physical properties of vegetable oils before their use as biofuel, so that the transesterification process of oils and fats is currently developed to obtain a noticeable lowering of viscosity in oils to employ the resulting product as biofuel in current existing diesel engines. Therefore, the accurate characterization of this parameter is essential to evaluate the result obtained in the process of ethanolysis.

Viscosity values were determined in an Oswald Proton Cannon-Fenske Routine Viscometer 33200, size 150, capillary viscometer (Sigma-Aldrich, Madrid, Spain). This value is based on determining the time needed for a given volume of fluid passing between two points marked on the instrument. The kinematic viscosity is given by the ratio between the dynamic viscosity (h, in poise, g/cm s) and the density (r, in g/cm^3^) υ = h/r (in cm^2^/s or centistokes (cSt), mm^2^/s). Samples, previously centrifuged at 3500 rpm for 10 min and filtered at 50 °C, are immersed in a thermostatic bath at 40 °C for 15 min, making sure that the temperature is stable. Then, samples are introduced into the viscometer and this, in turn, in the water bath, making sure that it is rigorously positioned vertically, with the bottom end at a minimum distance of 2 cm from the floor of the bath [31,32,33,40,42,43,44,45,46].

## 5. Conclusions

The results obtained in this study show the viability of a new paradigm related to the application of enzymes in fine chemistry, which consists of using scarcely purified enzymatic extracts, probably containing even mixtures of several enzymes, instead of very purified enzymes, as so far is usually done. The presence of other enzymes, together with that required for the desired process, should not affect this reaction, since these additional enzymes present do not encounter in the reaction medium other substrates to react with. Currently, only highly purified enzymes are used, since these enzymes are the only ones commercially available because they are those necessary for research in biochemistry. However, the high prices of these highly purified enzymes make them prohibitive for their application in organic synthesis or fine chemistry.

Therefore, to overcome the inconvenience of the high cost of the currently employed purified commercial enzymes, it would be advisable to study obtaining and testing the use of scarcely purified enzymatic extracts, obtained from standard strains of the same species of commercial lipases currently applied in the investigated process. This could be an appropriate method to drastically reduce the price of enzymatic biocatalysts, allowing the economic viability of biotechnological processes. Therefore, it would be advisable to approach research carried out in collaboration with a specialist in microbiology, to test the behavior of poorly purified enzymatic extracts, and to contain active enzymes in the synthesis processes of different molecules of interest, because the economic viability of biotechnological processes requires affordable biocatalysts, and this may be an appropriate method to drastically reduce the price of enzymatic biocatalysts.

## Figures and Tables

**Table 1:** Comparison of enzymatic technology versus chemical (alkaline and acid) technology for biodiesel production.

Parameter	Enzymatic Process	Chemical Process	
		Alkaline Process	Acid Process
**FFA content in the raw material**	FFA are converted to biodiesel	Soaps formation	FFA are converted to biodiesel
**Water content in the raw material**	It is not deleterious for lipase	Soaps formation. Oil hydrolysis resulting more soaps	Catalyst deactivation
**Biodiesel yield**	High, usually around 90%	High, usually >96%	High yields (>90%) only for high alcohol to oil molar ratio, high catalyst concentration and high temperature
**Reaction rate**	Low	High	Slower than for alkaline process
**Glycerol recovery**	Easy, high grade glycerol	Complex, low grade glycerol	Complex, low grade glycerol
**Catalyst recovery and reuse**	Easy Reusability proved but not sufficiently studied.	Difficult; neutralized by an acid Partially lost in post-processing steps	Difficult, the catalyst ends up in the by-products No reusable catalyst
**Energy costs**	Low Temperature: 20–50 °C.	Medium, Temperature: 60–80 °C.	High Temperature: >100 °C
**Catalyst cost**	High	Low	Low High cost of equipment due to acid corrosion
**Environmental impact**	Low; wastewater treatment not needed.	High; wastewater treatment needed.	High; wastewater treatment needed

**Table 2:** Schematic comparison of the main characteristics of the different technologies available to produce renewable liquid fuels, from vegetable oils.

Type	Biodiesel EN 14214	Biodiesel-Like Biofuels		
Name	Biodiesel	Gliperol^®^	DMC-Biod^®^	Ecodiesel^®^
**Reactive**	Methanol or Ethanol	Methyl acetate	Methyl carbonate	Ethanol
**Catalyst**	NaOH or KOH	Acid, Basic or Lipases	Basic or Lipases	Lipases
**Products**	3 FAME or 3 FAEE	glycerol triacetate + 3 FAME	Fatty acid Glycerol Carbonate + 2 FAME	1 Monoglyceride + 2 FAEE
**Byproducts**	Glycerol	no waste	no waste	no waste
**separation process & cleaning**	Complex	not needed	not needed	not needed
**Investment facilities**	Medium	Low	Low	Low
**Free fatty acids and/or water in the starting oil**	Free fatty acids are Transformed to soaps.	Free fatty acids are transformed to biofuel	Free fatty acids are transformed to biofuel	Free fatty acids are transformed to biofuel
**Catalyst cost**	Low	High	High	High
**Environmental impact**	High. Alkaline and saline effluents are generated. Wastewater treatment is needed.	Low	Low	Low

**Table 3:** Transesterification reaction yields obtained operating under standard reaction conditions: 6 mL of sunflower oil, 1.75 mL of ethanol, 30 °C, 25 μL of 10 N NaOH solution, higher stirring than 300 rpm, during 24 h of reaction time. As a biocatalyst is applied 0.5 g of enzymatic extract of standard CA strain from the CECT, obtained from several broths media obtained as it is indicated in Figure 2. The results obtained when repeating the reactions are presented. Viscosity (cSt) values of different reaction products obtained are also indicated.

Reaction	Biocatalyst	Viscosity (cSt)	Selectivity (%)	Conversion (%)
**Raw material**	**Sunflower oil**	34.5	3.5	33.3
**Blank samples**	**BLANK ^1^**	13.4	16.2	28.8
	**BLANK-YPD ^2^**	14.6	26.9	29.0
	**BLANK-YPD ^2^**	12.3	33.1	34.5
**1**	**YPD**	9.9	76.9	82.9
	**YPD-TB**	11.6	63.1	76.9
	**YPL**	11.6	61.1	72.6
	**YPL-TB**	12.4	42.1	44.7
**2**	**YPD**	10.8	72.1	73.5
	**YPD-TB**	11.9	67.0	71.8
	**YPL**	11.1	60.6	69.2
	**YPL-TB**	12.7	45.3	52.7

^1^ Reaction without biocatalyst; ^2^ An extract obtained from a YPD broth medium without inoculum or growth of the microbial strain is applied as a blank biocatalyst.
